# The Abandoned Trials of Pre-Exposure Prophylaxis for HIV: What Went Wrong?

**DOI:** 10.1371/journal.pmed.0020234

**Published:** 2005-07-19

**Authors:** Jerome A Singh, Edward J Mills

## Abstract

The dramatic protests that shut down trials of tenofovir as pre-exposure prophylaxis against HIV may prove damaging in our fight against the HIV pandemic.

New approaches to HIV/AIDS prevention are urgently needed to stem the estimated 5 million new infections that occur worldwide each year. One such promising, novel intervention has been the proposed use of the oral antiretroviral drug tenofovir (Viread) as a pre-exposure prophylaxis (PREP) in high-risk groups (for example, uninfected women who have high-risk commercial sex). However, emerging opposition has halted the progress of at least two important clinical trials of tenofovir as PREP and brought negative attention to tenofovir, somewhat similar to that visited on thalidomide more than four decades ago. This could prove damaging in the long term.

If tenofovir is someday proven to be clinically efficacious as a PREP, today's irresponsible reporting and activism surrounding tenofovir could cause those in need to snub the drug if, or when, it becomes licensed for use as a PREP. This unfortunate prospect raises questions about responsible media reporting, responsible conduct on the part of investigators and activists, and what should be done to avert or repair damaging trial-related disputes in the future.

## Protests against Trials of PREP

In July 2004, increasing pressure from activist groups and affiliated non-governmental organizations persuaded the Cambodian Prime Minister to halt the initiation of a PREP trial of tenofovir among Cambodian commercial sex workers [1], a trial funded by the United States National Institutes of Health and the Bill and Melinda Gates Foundation. The Cambodian Ministry of Health has provided no official statement for its decision to halt the trial.

The dramatic protest against the Cambodian trial at the XV International AIDS Conference in Bangkok, Thailand, caught the world's media attention [2] and brought tenofovir to the forefront of the public's attention (Figures 1 and 2). The primary reasons cited for the demonstrations included alleged inadequate prevention counseling by the study investigators, a lack of pre- and post-test HIV counseling, and the nonprovision of medical services and insurance for those who seroconverted during the study or experienced adverse events related to the trial drug [3]. Participant activist groups also argue that the safety of tenofovir for long-term use by individuals who are HIV negative has not been established, and that there was limited involvement of the target communities in the trial design [1,3,4]. The activist groups representing the participants argue that the participants take all of the risks and get little of the benefits.

**Figure 1:**
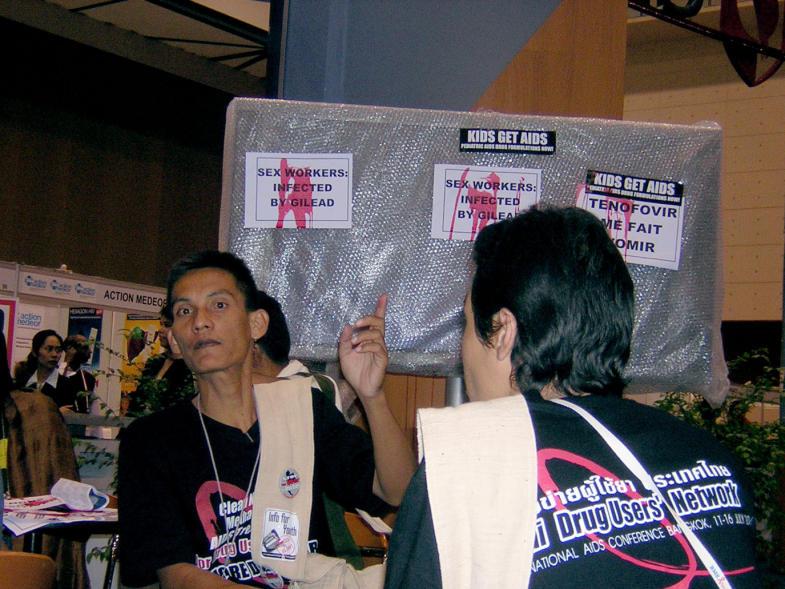
The Dramatic Protest at the Gilead Booth at the International AIDS Conference 2004 Caught the World's Attention (Photo: Act Up–Paris)

**Figure 2:**
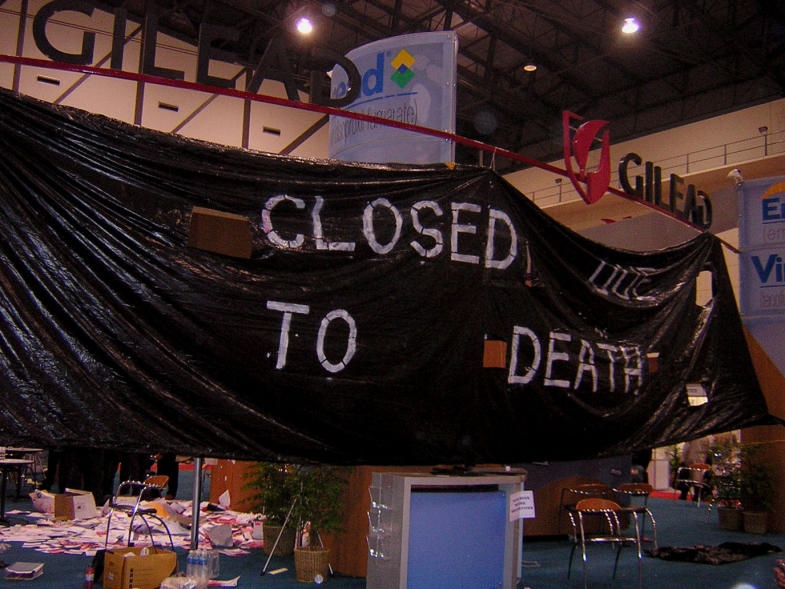
The Protest Closed the Gilead Booth at the International AIDS Conference 2004 (Photo: Act Up–Paris)

More recently, in February 2005, a similar trial in Cameroon, led by Family Health International (FHI), was halted by the Minister of Public Health. The activist group Act Up–Paris (www.actupparis.org) has led protests against both the Cambodian and Cameroonian trials, and has attracted French media attention. Act Up–Paris is supportive of tenofovir trials in general but has strong concerns about the current trials. In a documentary aired on French TV-2 and subsequent media reports, activists alleged that the FHI investigators intentionally allowed participants to become infected and provided inadequate counseling by having only five counselors for 400 participants [5].

Protest should be carried out in a responsible manner.

An independent inquiry, commissioned by the Cameroon Ministry of Public Health, reported on February 23, 2005, that they now require more regular reporting and a formal study site accreditation for a satellite hospital clinic [6]. The committee also confirmed that, contrary to popular belief and widespread reports, participants in the trial were not injected with HIV, and the study's tablets did not contain HIV. The inquiry recommended that the clinical trials could resume if the sponsors rectified certain administrative issues that the commission identified. The enrolled participants are being followed up, but neither tenofovir nor placebo is being dispensed.

On March 11, 2005, FHI made the announcement that the Nigerian arm of the tenofovir PREP trial will discontinue prematurely. FHI closed the trial voluntarily, because it determined that the study team was unable to comply with the required operational and laboratory procedures at the level necessary for conducting this study. The ability to meet these standards is critical for ensuring the safety of participants and the quality of the data from the study. This decision was made in conjunction with FHI's external, independent Data and Safety Monitoring Committee. More than 100 participants had been randomized. The announcement came as a disconcerting blow to the already fragile network of trials.

## The Protestors' Concerns

Activists and ethicists argue about the contentious issue of the standard of care in randomized trials [7]. According to the FHI protocols, participants who seroconverted during the trial would be provided with state-of-the-art antiretroviral therapy, if indicated (according to the World Health Organization's criteria for AIDS), with the possibility of extending treatment after the trial ended. Activist groups argue that treatment should be initiated in the same manner as would be provided in developed countries. Gilead, the maker of tenofovir, has promised to provide the drug at cost to the participating countries.

Guideline 29 of the Helsinki Declaration states that interventions should be tested against the best prophylactic interventions available [8]. In participants with a high risk of infection through sexual behaviour, this entails the provision of safe-sex education and condoms. The trial sites appear to have provided adequate counseling and male condom provision, but should also ensure female condom provision. Activists argue that if the primary endpoint is infection, counseling on safe sexual behaviour reduces the likelihood of finding an effect. They allege that investigators have a conflict of interest between meeting standards of human rights and obtaining scientific data.

In response, investigators claim that the tenofovir PREP trials were developed collaboratively with the host countries to meet relevant ethical standards. In West Africa, formative research studies with the community of participants helped to design the informed-consent instrument, to identify the preferred sites of receiving health care, and to identify sources of stigma, which the investigators tried to reduce.

Act Up–Paris and other activist groups report that they plan to continue protests against other tenofovir trials taking place elsewhere in the world (Table 1). However, while freedom of expression is a cherished ideal, we believe that protest should be carried out in a responsible manner. Important risks exist in all clinical trials, and the protection of research participants is of utmost concern. Speculation, unwarranted criticism, overreaction, or sensationalizing facts risk stigmatizing tenofovir and could jeopardize future attempts to find an efficacious PREP. This is in nobody's interest.

**Table 1:**
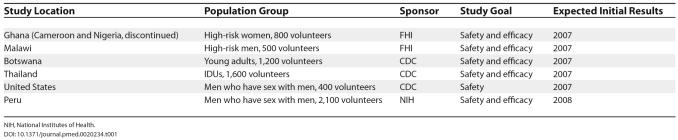
Current Tenofovir PREP Studies

NIH, National Institutes of Health.

## Ongoing Threats to PREP Trials

As of March 2005, there are at least six ongoing or planned human clinical trials of tenofovir as PREP (Table 1). The repercussions of the aforementioned trial closures will conceivably influence other current or proposed tenofovir PREP trials and could also detract from the use of tenofovir as a treatment agent. Act Up–Paris contends that the US-based trial will only examine safety, while the trials in developing countries examine efficacy.

Recent protests by the Thai Drug Users Network and other Thai AIDS advocacy groups, opposing the recent approval of a tenofovir prophylaxis trial funded by the US Centers for Disease Control and Prevention (CDC) among intravenous drug users (IDUs), should be setting off alarm bells for the trial investigators concerned. Thai activists cite ethical flaws in the trial design and a lack of community involvement on the part of the trialists. The activists argue that withholding the provision of clean injection equipment is an ethical violation, saying that clean equipment is a standard prevention tool akin to condoms, which are offered to trial participants in other countries who are chosen for their high-risk sexual behaviours.

But according to a CDC fact sheet, participating IDUs will be offered follow-up in a methadone drug-treatment program, and will receive bleach and instructions on how to use it to clean needles [9]. Consistent with Thai government policy and US national policy, sterile syringes will not be provided, although they are widely available in Thailand without a prescription and at low cost. Thailand has no harm-reduction policies for IDUs, and the government's war on drugs has been widely blamed for widespread human rights abuses against IDUs [10].

## There Is No Time to Waste

HIV/AIDS advocacy and other activist groups have openly criticized those involved in protesting these trials for what was seen as callous behaviour on their part. Most investigators and advocates for patients with HIV/AIDS laud the past work of activist groups in attracting the world's attention to the HIV epidemic. Indeed, activism can undoubtedly play an important role in ensuring that researchers and sponsors maintain ethical standards. However, activism should be based on informed opinion and communication.

Activism should be based on informed opinion.

In May 2005, the Bill and Melinda Gates Foundation brought together activist, advocacy, and research groups to discuss future tenofovir trials. In an effort to engage stakeholders, the meeting sought resolution and clarifications to the standards of care and prophylaxis in planned provision. The meeting identified the many rumors and miscommunications that had existed in the reporting of the closed trials. It exemplified the necessity for early interventions to promote communication, in order to prevent partisan behaviour among stakeholders.

The rapidly collapsing tenofovir trial network shows that a lack of communication between activists, participants, and researchers can lead to suspicion, speculation, and, ultimately, damaging outcomes. While clinical trial investigators have increasingly begun to involve stakeholder groups in medical decision-making and trial planning, this gesture must go beyond mere tokenism. Investigators should engage in pre-trial “preventative diplomacy”. This celebrated dispute resolution mechanism is often used in political contexts but could find useful application in the research arena, too. While anything intended to keep a conflict from worsening might be described as “preventative”, preventative diplomacy involves “proaction” rather than reaction and emphasizes that crises can be better addressed before or as they emerge rather than when they have already deepened and widened.

Instruments that may be used to prevent the emergence or escalation of disputes culminating in trial suspension include the following: (1) the establishment of early warning mechanisms (such as a community liaison officer), (2) fact finding missions (these may establish that the operational reality does not resonate with protocol schedule), (3) confidence-building measures (such as the inclusion of activist groups in community advisory boards), (4) engaging the media, and (5) education (particularly on important issues such as therapeutic misconception, compensation for study-related injuries, and post-trial benefits). The development of a forum for identifying mutual interests and concerns, while still invoking reciprocity and transparency, may identify early concerns.

The investigators from the PREP trials report that they did involve activist and advocacy groups in designing the trials, but say that they were unsuccessful in addressing the wider activist community. Investigators should not merely encourage the involvement of activist groups in future prevention trials—they must make a genuine attempt to address their concerns. Such an approach may have averted the trial closures.

The world desperately needs efficacious HIV prevention and therapy. If tenofovir is shown to be an effective PREP agent, it will become a powerful tool in the fight against AIDS, but it will need to be delivered alongside behavioural interventions, condoms, clean needles, HIV testing, and access to HIV treatment. The ethics of the aforementioned Cambodian and Cameroonian tenofovir trials will likely remain forever contentious. The operations of the Nigerian trial remain a continuing issue, concerning how to assure research quality meets international standards in resource-poor settings. However, the important lesson to be learned from these experiences is that investigators, sponsors, participants, members of the study community, government authorities, and activist groups must actively engage at all stages of a trial to ensure that the study is conducted in a manner that is beneficial to, and respectful of, the participants, while remaining scientifically sound.

Stakeholders must rise above ideological differences and keep their eye on the ultimate goal: combating AIDS. If disputes arise, they must meaningfully commit themselves to addressing the issues expeditiously and in a manner that is conducive to ongoing dialogue and a sustainable relationship. A failure to do otherwise will frustrate attempts to combat AIDS and needlessly prolong the suffering of those in need.

## Abbreviations

CDC: Centers for Disease Control and Prevention
FHI: Family Health International
IDU: intravenous drug user
PREP: pre-exposure prophylaxis
